# 

**Published:** 1864-11

**Authors:** 


### CONTENTS.

### ORIGINAL CONTRIBUTIONS.

ART. XXXVII. Some Account of the Prevailing Epidemic in the Northwest, variously designated, but usually popularly denominated "Spotted Fever." By J. Adams Allen, M.D., &c.... 593

ART. XXXVIII. Conservative Surgery. Cases of Resection. Reported by N. W. Abbott, late Surgeon of the 80th Regt. Ill. Vols.,..... 609

ART. XXXIX. Cases Read before the Chicago Medical Society. By Hiram Wanzer, M.D., of Chicago, ........ 612

ART. XL. Introductory Lecture. By N. S. Davis, M.D., Chicago,.. 616

### SELECTIONS.

The Medical Management of Insane Women. By Horatio Robinson Storer, M.D., ........ 626

On Neuralgic Affections following Injuries of Nerves. By J. Mason Warren, M.D., Surgeon at the Massachusetts General Hospital,........ 636

Letter from Dr. W. N. Cote, ........ 646

### BOOK NOTICES.

The Functions and Disorders of the Reproductive Organs in Childhood, Youth, Adult Age, and Advanced Life considered in their Physiological, Social, and Moral Relations. By William Action, M.R.C.S., ...... 649

Therapeutics and Materia Medica. By Alfred Stilte, M.D.,........ 650

The Book of Prescriptions. By Henry Beasley,........ 650

Diphtheria. Its Nature and Treatment. By Daniel Slade, M.D.,........ 650

Lectures on Venerial Diseases. By Wm. A. Hammond, M.D., ........ 650

### EDITORIAL.

Galactogogues,........ 651

The Illinois State Medical Society. Committee on Diseases of the Eye,..... 651

City Mortality.—Statistics for November,........ 652

Letter from the Permanent Secretary of the American Medical Association, 653

Catheterism of the Duodenum and Jejunum, ........ 653

Obstinate Constipation,........ 654

Professor Trousseau on Aphasia, ........ 654

